# A Simplified and Solvent-Reduced HPLC-UV/Vis Method for the Simultaneous Determination of Major Marker Compounds in *Rhodiola rosea* L.

**DOI:** 10.3390/molecules31203734

**Published:** 2026-10-18

**Authors:** Alexandra Stabl, Katalin Reich, Ivonne Wittmann, Sabine Wittmann, Özlem Özmutlu Karslioglu, Sabine Grüner

**Affiliations:** 1 Department of Bioengineering, University of Applied Sciences Weihenstephan-Triesdorf, Am Hofgarten 10, 85354 Freising, Germany; 2 Institute of Horticulture, University of Applied Sciences Weihenstephan-Triesdorf, Am Staudengarten 8, 85354 Freising, Germany; ivonne.wittmann@hswt.de; 3 Digital Agriculture, HEF World Agricultural Systems Center, Department of Life Science Engineering, TUM School of Life Sciences, Technical University of Munich, 85354 Freising, Germany; sabine.wittmann@tum.de; 4 Institute of Food Technology, University of Applied Sciences Weihenstephan-Triesdorf, Am Staudengarten 11, 85354 Freising, Germany; oezlem.oezmutlu@hswt.de

**Keywords:** *Rhodiola rosea* L., salidroside, rosavins, acetonitrile, ethanol extraction, HPLC-UV/Vis, method validation

## Abstract

*Rhodiola rosea* L. is traditionally used to relieve stress-related symptoms and has been investigated for its potential effects on depression and anxiety. The present study developed and validated a solvent-reduced HPLC-UV/Vis method for the determination of characteristic secondary metabolites in *R. rosea*. To minimize solvent consumption and eliminate methanol from sample preparation, extraction with 40% (*v*/*v*) ethanol was combined with a simplified chromatographic protocol. Extracts were analyzed by HPLC-UV/Vis for the target analytes salidroside, tyrosol, rosarin, rosavin, and rosin. Chromatographic separation was achieved within 35 min using a water–acetonitrile gradient at a flow rate of 0.8 mL min^−1^. Compared with the reference method described by Ganzera et al., the optimized protocol reduced the total analysis time from 44 to 35 min and eliminated the post-run methanol washing step. Method validation yielded analyte-specific recoveries ranging from 102 ± 3% to 114 ± 4% and relative standard deviations from 0.5% to 8.0%. The limits of quantification were 0.497, 0.302, 0.477, 0.148, and 0.189 µg mL^−1^ for salidroside, tyrosol, rosarin, rosavin, and rosin, respectively. Quantification was performed by external standard calibration. The developed method offers a simplified, solvent-reduced approach for the quantitative determination of major *R. rosea* marker compounds, thereby reducing both analysis time and solvent consumption.

## 1. Introduction

*Rhodiola rosea* L. (*R. rosea*) is a perennial medicinal plant belonging to the family Crassulaceae and occurs naturally in Arctic and sub-Arctic regions of Europe and Asia, including China, Mongolia, Russia, and Scandinavia [1,2]. The plant exhibits adaptogenic properties primarily attributed to its characteristic phenolic secondary metabolites [2]. In addition, antioxidant and antidepressant activities have been reported, supporting both its traditional and current therapeutic applications [3,4].

The roots and rhizomes are of particular pharmacological relevance, as they contain high concentrations of bioactive constituents. Extracts derived from these plant organs have been associated with stress-relieving, antidepressant, and immunomodulatory effects. The European Medicines Agency recognizes preparations derived from *R. rosea* roots and rhizomes as traditional herbal medicinal products for the temporary relief of stress-related symptoms, such as fatigue and weakness [5]. In the European Union, *R. rosea* root preparations are marketed as food supplements, although the regulation of botanicals in foods is not fully harmonized across Member States [6]. Several studies have also reported stimulatory effects on the central nervous system, which may contribute to increased attention and improved cognitive performance [4,7]. Based on these observations, *R. rosea* has attracted interest as a potential supportive approach to age-related cognitive decline [7,8]. 

More than 150 biologically active compounds have been identified in *R. rosea*, including phenylethanoids, phenylpropanoids, flavonoids, monoterpenoids, and triterpenes [9]. Among these compounds, salidroside, tyrosol, rosarin, rosavin, and rosin are regarded as important marker compounds and are commonly used to assess the quality of plant materials and commercial preparations [9,10]. Salidroside and its biosynthetic precursor, tyrosol, belong to the class of phenylethanoid derivatives and are known for their antioxidant activity. Rosarin, rosavin, and rosin, collectively referred to as rosavins, are classified as phenylpropanoid derivatives of cinnamyl alcohol and exhibit neurostimulatory and antioxidant properties [11,12]. 

Commercial *R. rosea* extracts are commonly characterized using rosavins and salidroside as analytical marker compounds. Specifications of approximately 3% total rosavins and 0.8–1.0% salidroside have been reported for standardized extracts, primarily to support compositional consistency and quality control [11]. Reliable analytical methods are therefore required for the qualitative and quantitative determination of these marker compounds. Ganzera et al. developed the first HPLC method for the simultaneous determination of salidroside, rosarin, rosavin, rosin, and rosiridin in a single chromatographic run [10]. The method employed methanolic extraction, a phosphate buffer–acetonitrile gradient system, and a post-run washing step using phosphoric acid in methanol. It met the key validation criteria and enabled the reliable identification and quantification of major marker compounds in *R. rosea* plant material and commercial products.

Although the method developed by Ganzera et al. provides reliable chromatographic separation and quantification of major *R. rosea* marker compounds, it relies on methanol for both sample preparation and chromatographic washing. In contrast, ethanol–water mixtures are commonly used to produce *R. rosea* extracts and commercial herbal preparations. Aqueous ethanol mixtures, including 40% (*v*/*v*) ethanol, have been investigated for the extraction of salidroside and rosavins from *R. rosea* roots and rhizomes [13].

In addition to solvent-related considerations, increased analytical throughput and reduced resource consumption are increasingly important in routine quality-control laboratories. Accordingly, this study adapted and optimized an HPLC-UV/Vis method for the simultaneous determination of five major marker compounds in *R. rosea*: salidroside, tyrosol, rosarin, rosavin, and rosin. The primary objective was to reduce solvent consumption and resource requirements while maintaining analytical performance.

Based on the method described by Ganzera et al., the methanol-based extraction procedure was replaced with extraction using 40% (*v*/*v*) ethanol. Furthermore, the chromatographic protocol was simplified by reducing the flow rate and eliminating the post-run washing step. Alternative methods reported in the literature were experimentally evaluated in preliminary method-transfer trials [14,15]. However, none of these methods could be successfully established under the available laboratory conditions while maintaining the chromatographic performance required for the simultaneous determination of all five target analytes. Consequently, the optimization focused on modifying the established method of Ganzera et al. while maintaining adequate separation and quantification of the target analytes [10].

The analytical performance of the optimized method was subsequently assessed for chromatographic separation, linearity, precision, accuracy, limit of detection (LOD), and limit of quantification (LOQ). While maintaining satisfactory analytical performance, the modified method reduced solvent consumption by approximately 16 L per 1000 analyses, corresponding to savings of approximately 2 L of acetonitrile and 14 L of water. Furthermore, the shorter run time resulted in a cumulative reduction in analysis time of approximately 150 h per 1000 analyses.

In summary, the optimized method provides a practical, solvent-reduced, and resource-efficient approach for the routine determination of *R. rosea* marker compounds in phytochemical quality control.

## 2. Results and Discussion

### 2.1. HPLC-UV/Vis Analysis

#### 2.1.1. Secondary Metabolites

**Figure 1:**
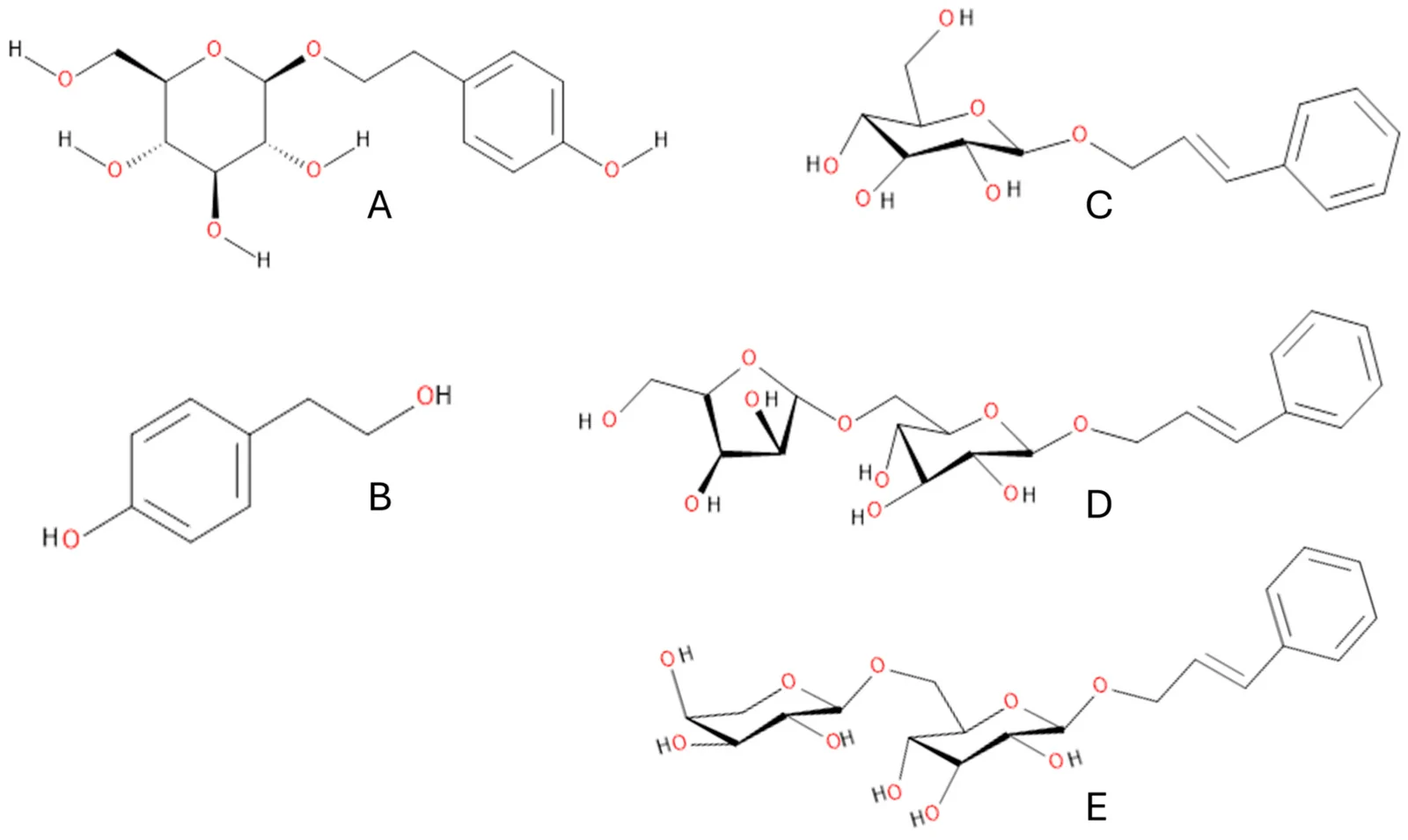
Chemical structures of the investigated *R. rosea* marker compounds: (**A**) salidroside, (**B**) tyrosol, (**C**) rosin, (**D**) rosarin, and (**E**) rosavin.

The major marker compounds investigated in this study were salidroside, tyrosol, rosarin, rosavin, and rosin [16]. The chemical structures of these compounds are shown in Figure 1. 

Rosarin, rosavin, and rosin belong to the class of phenylpropanoid derivatives and are considered characteristic marker compounds of *R. rosea* [17]. These constituents have been reported to exhibit antioxidant and neurostimulatory activities. Salidroside is the glycosidic form of tyrosol, and both compounds are classified as phenylethanoid derivatives [12]. In addition to its antioxidant activity, salidroside has been associated with antidepressant and anxiolytic effects, which may contribute to the adaptogenic profile of *R. rosea* [1,6]. The analytes investigated are major marker compounds commonly used to characterize and assess the quality of *R. rosea* preparations. In addition to salidroside and tyrosol, rosavin and rosin are regarded as key marker compounds and have been associated with the biological activity of *R. rosea* [9].

**Figure 2:**
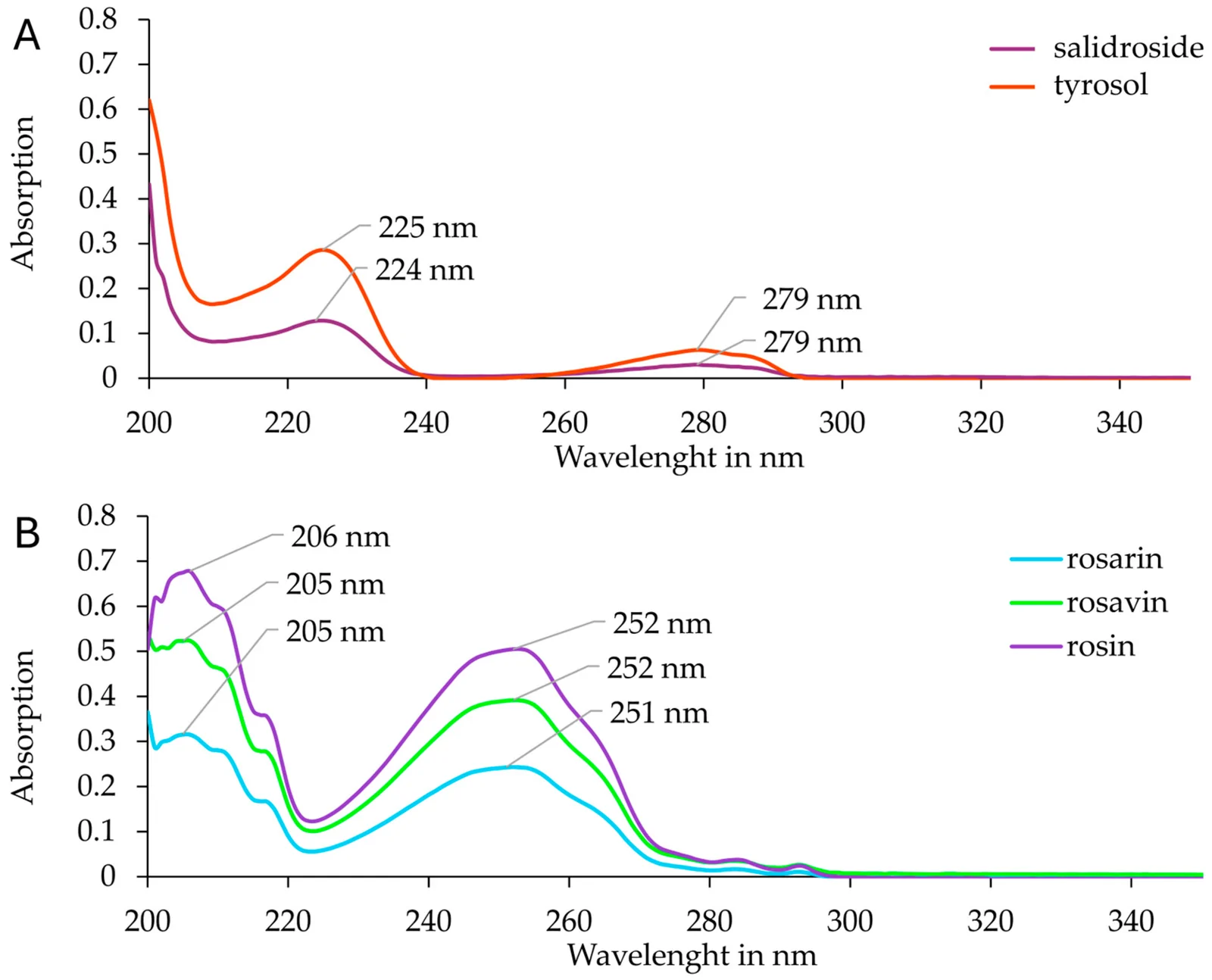
UV/Vis spectra of (**A**) salidroside and tyrosol and (**B**) rosarin, rosavin, and rosin.

UV/Vis spectra were recorded for each investigated secondary metabolite to determine a common detection wavelength suitable for simultaneous quantification (Figure 2). 

The rosavins, namely rosarin, rosavin, and rosin, exhibited absorption maxima at approximately 205 nm, whereas salidroside and tyrosol showed absorption maxima at approximately 225 nm. Comparable absorption maxima have been previously reported [14,18]. Despite this difference, salidroside and tyrosol exhibited sufficiently strong absorbance at 205 nm. Although this wavelength does not correspond to their individual absorption maxima, the signal intensity remained adequate for reliable detection and quantification. Consequently, 205 nm was selected as a compromise wavelength, enabling the simultaneous detection of all five target analytes within a single chromatographic run. The use of a single detection wavelength is advantageous for routine quantitative analysis because it enables simultaneous monitoring of all target compounds without changing the detector wavelength during chromatographic separation. The use of 205 nm for the simultaneous determination of *R. rosea* marker compounds has also been described in the literature, further supporting its suitability for multianalyte analysis [10,18].

#### 2.1.2. Method Details

The HPLC-UV/Vis method and extraction procedure were adapted from the method described by Ganzera et al. [10]. To optimize the method, the flow rate was reduced to 0.8 mL min^−1^, and the additional washing step with 0.1% phosphoric acid in methanol was omitted (Table 1).

The mobile phase consisted of acetonitrile and water. The acetonitrile proportion was increased from 10% to 20% over 30 min at a constant flow rate of 0.8 mL min^−1^. The initial conditions were then restored, resulting in a total run time of 35 min.

Compared with the original method described by Ganzera et al., the modified protocol reduced the flow rate from 1.0 to 0.8 mL min^−1^ and eliminated the methanol-based washing step after each injection. In the original method, a washing step was introduced to remove matrix components and to prevent an increase in column backpressure caused by the high tannin content of *R. rosea* extracts [10]. In the present study, omitting this washing step did not preclude chromatographic analysis of the target compounds and simplified the analytical workflow. During routine application of the method over approximately two years and 2200 chromatographic runs, no noticeable increase in column backpressure, loss of chromatographic performance, or unusual wear of either the guard column or the analytical column was observed.

**Figure 3:**
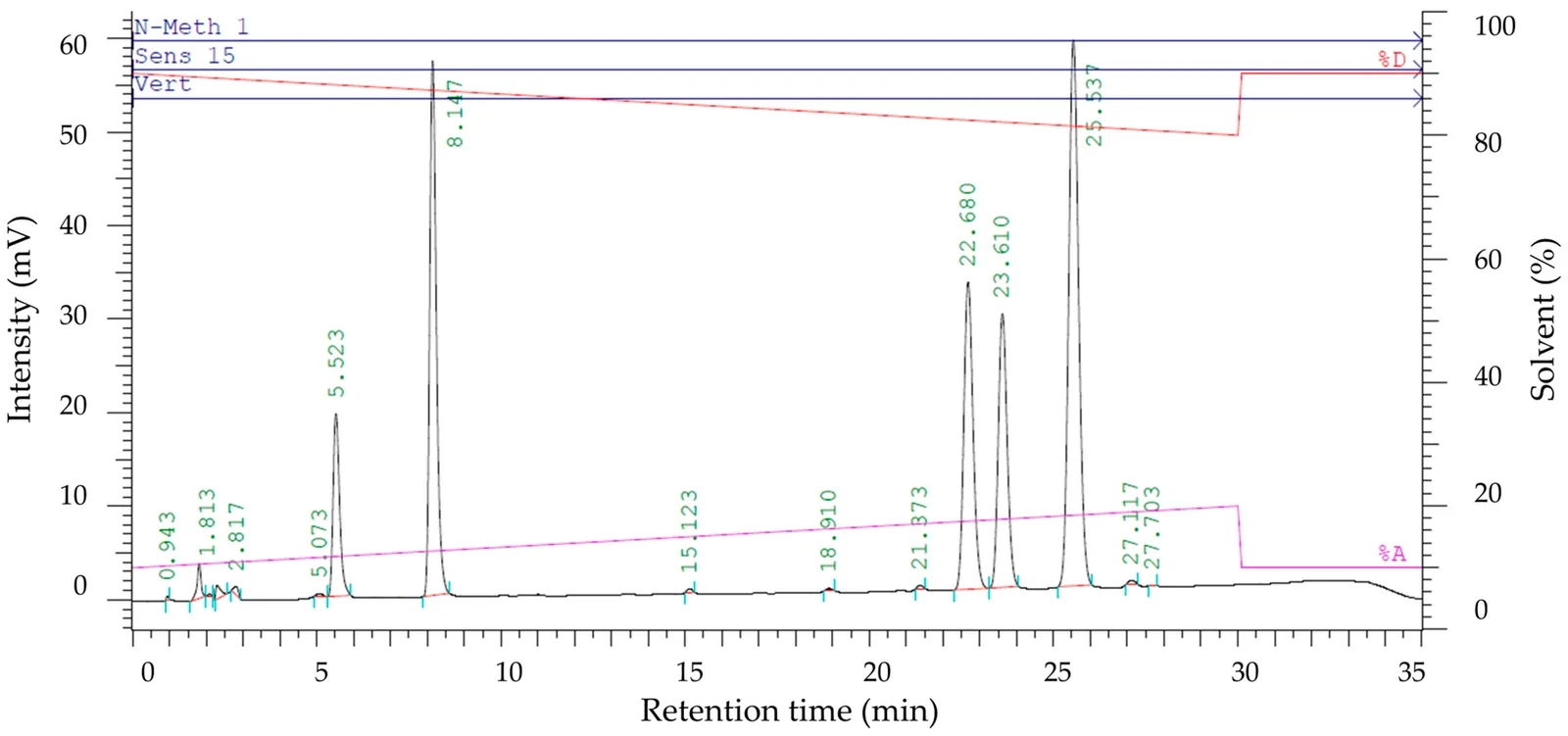
Representative chromatogram of the mixed reference standard containing salidroside, tyrosol, rosarin, rosavin, and rosin. The corresponding retention times were 5.523, 8.217, 22.680, 23.610, and 25.507 min, respectively.

A representative chromatogram of the mixed reference standard is shown in Figure 3. A chromatogram of a higher-concentration mixed reference standard is provided in Supplementary Figure S1 to illustrate the separation and peak shapes of rosarin and rosavin under increased analyte loading.

All five target analytes were detected in a single chromatographic run. As reported previously, the chromatographic separation of *R. rosea* marker compounds is challenging because salidroside exhibits substantially higher polarity than the rosavins, whereas rosarin, rosavin, and rosin differ primarily in their sugar moieties [10,14,18]. Nevertheless, the target analytes could be assigned based on their retention times and comparison with authenticated reference standards.

**Figure 4:**
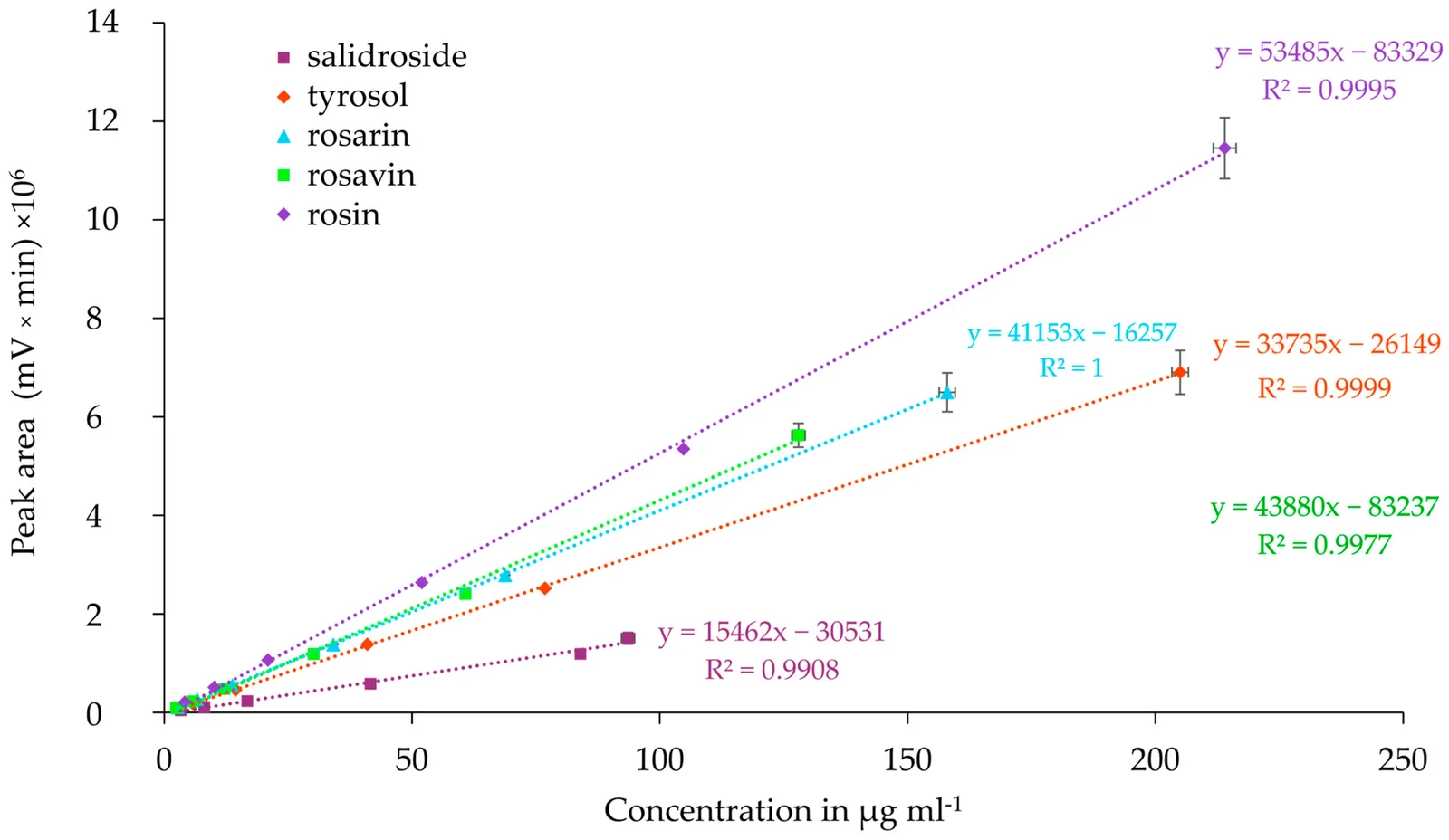
Calibration curves of the five target analytes based on standard solutions. Data points represent mean peak areas, and error bars indicate the standard deviation of three replicate measurements (*n* = 3).

Calibration curves were constructed using the external standard method. Reference standards of salidroside, tyrosol, rosarin, rosavin, and rosin were analyzed at different concentration levels under identical chromatographic conditions. The resulting detector responses, expressed as peak areas, were used to construct individual calibration curves for the quantitative determination of the analytes in the plant extracts. The calibration curves are presented in Figure 4.

**Figure 5:**
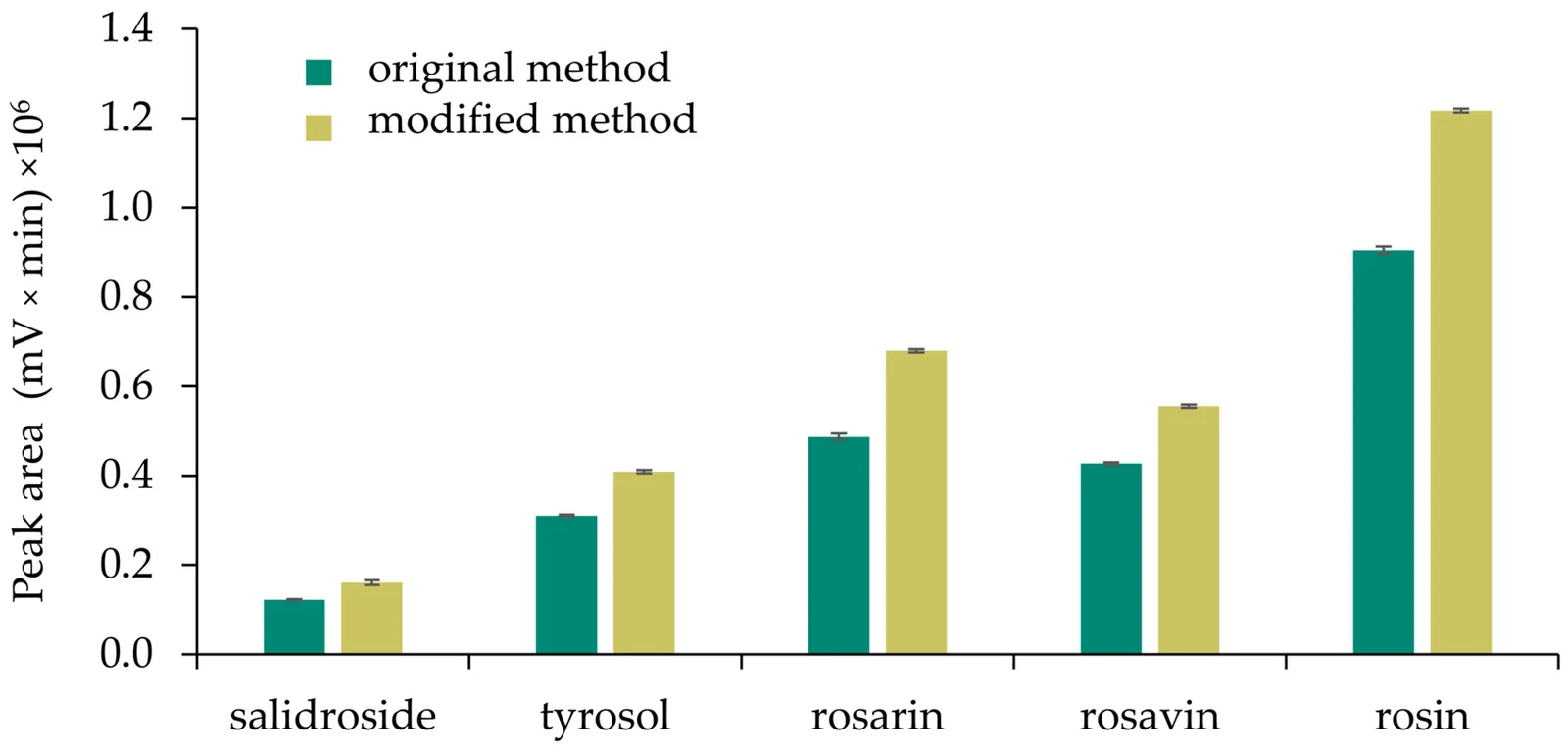
Comparison of the peak areas obtained using the original HPLC method reported by Ganzera et al. [10] (flow rate: 1.0 mL min^−1^, including an additional washing step) and the modified method developed in this study (flow rate: 0.8 mL min^−1^, without a post-run washing step). Data are presented as mean values (*n* = 3).

Compared with the method reported by Ganzera et al. [10], the modified chromatographic protocol maintained sufficient signal intensity for all investigated marker compounds while reducing methodological complexity by omitting the post-run washing step. The results indicate that the target analytes could be detected and quantified under the modified chromatographic conditions. Figure 5 compares the peak areas obtained using the original and modified methods. 

### 2.2. Method Validation

#### 2.2.1. Peak Resolution

To evaluate chromatographic separation, the resolution factor (*R*_s_*)* was used as a performance parameter. Attention was given to the separation of rosarin, rosavin, and rosin because of their structural similarity and comparable retention behavior. Chromatographic resolution (*R*_s_) was calculated using Equation (1), as described by Snyder, Kirkland, and Glajch [19], based on the difference between the retention times of two adjacent peaks and their corresponding baseline peak widths:

(1) $$R_{s}=2\times \frac{t_{r2}-t_{r1}}{w_{b1}+w_{b2}}$$

where *t*_r_ represents the retention time, and *w*_b_ represents the baseline peak width. The calculated resolution factors are summarized in Table 2. Compared with the original method described by Ganzera et al. [10], a similar resolution was obtained for rosarin and rosavin, whereas the resolution between rosavin and rosin was higher with the modified method. 

The comparable resolution values for rosarin and rosavin are consistent with their structural similarity. Rosarin, rosavin, and rosin differ primarily in their sugar moieties and therefore exhibit closely related chromatographic retention behavior [10]. Peak assignment was based on a comparison of the retention times with those of the authenticated reference standards.

The modified method reduced the analysis time from 44 to 35 min, corresponding to a reduction of approximately 20%. The resolution between rosavin and rosin increased from 1.10 ± 0.10 to 2.30 ± 0.06, whereas the resolution between rosarin and rosavin remained similar at 1.06 ± 0.01 and 1.08 ± 0.03, respectively. Thus, the shorter analysis time was achieved without a measurable loss of resolution for either of the two peak pairs investigated. However, complete baseline separation of rosarin and rosavin was not achieved.

#### 2.2.2. Limits of Detection and Quantification

To evaluate the sensitivity of the modified method, the limits of detection (LOD) and quantification (LOQ) were determined. Both parameters were calculated using Equations (2) and (3), respectively, based on the standard deviation of the regression analysis and the slope of the corresponding calibration curve [20]:

(2) $$\mathrm{LOD}=3.3\times \frac{\mathrm{SD}}{m}$$

(3) $$\mathrm{LOQ}=10\times \frac{\mathrm{SD}}{m}$$

where SD is the standard deviation of the regression analysis, and *m* is the slope of the respective calibration curve. The calculated LOD and LOQ values are summarized in Table 3. 

The LOD values ranged from 0.049 to 0.164 µg mL^−1^, whereas the LOQ values ranged from 0.148 to 0.497 µg mL^−1^. The lowest detection and quantification limits were obtained for rosavin, while salidroside showed the highest values. The observed differences in LOD and LOQ values indicate analyte-specific differences in analytical sensitivity under the selected chromatographic conditions. Nevertheless, all analytes had calculated LOQ values below 0.5 µg mL^−1^, indicating that the method provides sufficient analytical sensitivity for their quantification under the investigated conditions. The obtained sensitivity supports the method’s application to the routine analysis of the principal marker compounds in *R. rosea* extracts at concentrations relevant to phytochemical quality control.

#### 2.2.3. Precision and Accuracy

Precision was evaluated by repeated analysis of the same *R. rosea* root and rhizome extract under identical chromatographic conditions. The relative standard deviations (RSDs) obtained for salidroside, tyrosol, rosarin, rosavin, and rosin were 0.6%, 8.0%, 1.2%, 0.5%, and 1.8%, respectively. The RSD values were ≤1.8% for salidroside, rosarin, rosavin, and rosin, whereas tyrosol showed a higher RSD of 8.0%. These results indicate good repeatability for four out of the five investigated marker compounds and greater variability in the quantification of tyrosol.

Accuracy was assessed using recovery experiments. For this purpose, *R. rosea* analytes were analyzed at different times, and recovery rates were calculated using Equation (4).

(4) $$\mathrm{Recovery}\;(\%)=\frac{\mathrm{measured}\;\mathrm{concentration}}{\mathrm{expected}\;\mathrm{concentration}}\times 100$$

The recovery rates ranged from 102 ± 3% to 114 ± 4%. The analyte-specific recovery values were 114 ± 4% for salidroside, 102 ± 3% for tyrosol, 102 ± 3% for rosarin, 107 ± 4% for rosavin, and 104 ± 4% for rosin. Recoveries close to 100% were obtained for tyrosol, rosarin, rosavin, and rosin, whereas salidroside showed a positive bias. Ganzera et al. reported recoveries ranging from 97.49% to 100.88% for the corresponding marker compounds [10]. Overall, the recovery results support the accuracy of the modified method for tyrosol, rosarin, rosavin, and rosin, while the positive bias observed for salidroside should be considered when interpreting its quantitative results.

Collectively, the resolution, sensitivity, precision, and recovery data support the applicability of the modified method for quantitative determination of the principal *R. rosea* marker compounds under the investigated conditions. Although complete baseline separation was not achieved for the rosarin–rosavin peak pair, repeatability was demonstrated by RSD values of 1.8% or less for four analytes, while tyrosol exhibited greater variability with an RSD of 8.0%.

## 3. Materials and Methods

### 3.1. Plant Material

The plant material, consisting of *R. rosea* roots and rhizomes, was obtained from a cultivated field site in Thuringia, Germany. The plants were harvested in their fourth year of cultivation. The *R. rosea* roots and rhizomes were dried in an oven at 40 °C until a constant weight was reached. For extraction, 150 mg of the dried and finely ground *R. rosea* roots and rhizomes was weighed into a 15 mL polypropylene centrifuge tube (Carl Roth GmbH + Co. KG, Karlsruhe, Germany). Subsequently, 5 mL of 40% (*v*/*v*) ethanol in deionized water was added. The mixture was subjected to ultrasound-assisted extraction in an ultrasonic bath (Retsch GmbH, Haan, Germany) for 10 min to enhance the release of secondary metabolites. After sonication, the suspension was centrifuged at 3500 rpm for 5 min, and the supernatant was carefully collected. The remaining solid residue was re-extracted under identical conditions using fresh extraction solvent. The extraction procedure was performed three times in total. The combined extracts, with a final volume of 15 mL, were filtered through a 0.2 µm membrane filter (Carl Roth GmbH + Co. KG, Karlsruhe, Germany) and transferred to amber glass vials before HPLC analysis.

### 3.2. Instrumentation and Reagents

HPLC analyses were performed using a VWR Hitachi Chromaster system comprising a 5160 HPLC pump, a 5310 column oven, and a 5410 UV/Vis detector (VWR International, Darmstadt, Germany). Chromatographic separation was carried out on a ZORBAX Eclipse XDB-C18 column (150 × 4.6 mm, 5 µm particle size) equipped with a matching ZORBAX Eclipse XDB-C18 guard column (4.6 × 12.5 mm, 5 µm) (Agilent, Santa Clara, CA, USA). All solvents used for extraction and HPLC analysis, including HPLC-grade acetonitrile, ultrapure water, and ethanol (99%, *v*/*v*), were purchased from VWR Chemicals LLC. Certified reference standards of salidroside, tyrosol, rosarin, rosavin, and rosin were obtained from PhytoLab GmbH & Co. KG, Vestenbergsgreuth, Germany, and were prepared in 40% (*v*/*v*) ethanol as described in previous studies [10,21].

### 3.3. HPLC Method

The HPLC method was adapted from the procedure described by Ganzera, Yayla, and Khan [10] with minor modifications. Chromatographic separation was performed at a flow rate of 0.8 mL min^−1^ using HPLC-grade acetonitrile and water as the mobile-phase components. Gradient elution was performed by increasing the acetonitrile content from 10% to 20% over 30 min. The initial conditions were then restored and maintained until the end of the 35 min run. The column oven temperature was maintained at 30 °C throughout the analysis. Separation was performed using a ZORBAX Eclipse XDB-C18 column (150 × 4.6 mm, 5 µm particle size) protected by a matching ZORBAX Eclipse XDB-C18 guard column (4.6 × 12.5 mm, 5 µm) (Santa Clara, CA, USA). The secondary metabolites were detected at 205 nm using a UV/Vis detector, enabling the simultaneous monitoring of all target analytes. Pure ethanol was used as a negative control to verify the absence of interfering signals. A mixed standard solution containing known concentrations of salidroside, tyrosol, rosarin, rosavin, and rosin in 40% (*v*/*v*) ethanol was used as a positive control for peak identification and system suitability.

### 3.4. Validation

The modified HPLC method was validated with respect to linearity, precision, accuracy, LOD, and LOQ. The marker compounds in the *R. rosea* extracts were quantified by external calibration using authenticated reference standards of salidroside, tyrosol, rosarin, rosavin, and rosin.

#### 3.4.1. Linearity

The linearity of the detector response was evaluated for all five reference compounds over a concentration range of 2.5–200 µg mL^−1^. Standard solutions were prepared in 40% (*v*/*v*) ethanol. An individual calibration curve was constructed for each analyte. Linear regression analysis showed coefficients of determination (R^2^) of 0.9908 for salidroside, 0.9990 for tyrosol, 1.0000 for rosarin, 0.9977 for rosavin, and 0.9995 for rosin.

#### 3.4.2. Precision

The precision of the method was evaluated by repeated analysis of the same *R. rosea* sample obtained from outdoor cultivation. The sample was analyzed ten times under identical experimental conditions. The resulting relative standard deviations (RSDs) were 0.6% for salidroside, 8.0% for tyrosol, 1.2% for rosarin, 0.5% for rosavin, and 1.8% for rosin. These results demonstrated good precision for salidroside, rosarin, rosavin, and rosin, while tyrosol showed greater variability.

#### 3.4.3. Accuracy

Accuracy was assessed using recovery experiments. The *R. rosea* analytes were each analyzed at different times and the recovery rate was calculated using Equation (4).

#### 3.4.4. Limits of Detection and Quantification

LOD and LOQ were determined using pure standard solutions at concentrations ranging from 0.01 to 0.5 µg mL^−1^. Each concentration was analyzed in triplicate. LOD and LOQ were calculated using Equations (2) and (3), respectively.

## 4. Conclusions

The HPLC-UV/Vis method adapted from Ganzera et al. enabled the simultaneous determination of salidroside, tyrosol, rosarin, rosavin, and rosin in root and rhizome extracts of *R. rosea*. Replacing methanol with 40% (*v*/*v*) ethanol during sample preparation, reducing the flow rate to 0.8 mL min^−1^, and omitting the post-run methanol washing step reduced the total run time from 44 to 35 min. Based on the calculated consumption per 1000 analyses, these modifications reduced solvent use by approximately 16 L and the total analysis time by approximately 150 h.

The LOD and LOQ values calculated from the pure standard solutions were below 0.17 and 0.50 µg mL^−1^, respectively, for all five analytes. Repeatability was characterized by analyte-specific RSD values ranging from 0.5% to 8.0%, while recoveries ranged from 102 ± 3% to 114 ± 4%, with salidroside showing a positive bias. Although complete baseline separation was not achieved for the rosarin–rosavin peak pair, the target analytes could be assigned by comparison with authenticated reference standards and quantified reproducibly under the investigated conditions. Overall, the modified method provides a simplified, solvent-reduced approach for the routine determination of the investigated *R. rosea* marker compounds.

## Figures and Tables

**Table 1:** Gradient conditions used for the modified HPLC method.

Time (min)	Acetonitrile (%)	Water (%)	Flow Rate (mL min^−1^)
0.0	10.0	90.0	0.8
30.0	20.0	80.0	0.8
30.1	10.0	90.0	0.8
35.0	10.0	90.0	0.8

**Table 2:** Comparison of the resolution factors and analysis times of the original and modified methods.

Method	*R*_s_ Between Rosarin and Rosavin	*R*_s_ Between Rosavin and Rosin	Analysis Time (min)
Original method [10]	1.06 ± 0.01	1.10 ± 0.10	44
Modified method	1.08 ± 0.03	2.30 ± 0.06	35

**Table 3:** Limits of detection and quantification for the five investigated marker compounds.

	Salidroside (µg mL^−1^)	Tyrosol (µg mL^−1^)	Rosarin (µg mL^−1^)	Rosavin (µg mL^−1^)	Rosin (µg mL^−1^)
LOD	0.164	0.100	0.158	0.049	0.063
LOQ	0.497	0.302	0.477	0.148	0.189

## Data Availability

The original contributions presented in this study are included in the article/Supplementary Material. Further inquiries can be directed to the corresponding authors.

## Supplementary Materials

The following supporting information can be downloaded at: https://www.mdpi.com/article/10.3390/molecules31203734/s1, Figure S1: Chromatogram of a higher-concentration mixed reference standard: (A) complete chromatogram of the five target analytes and (B) enlarged view of the rosavin-containing region, highlighting the critical rosarin–rosavin peak pair. Analyte concentrations: salidroside, 84.0 μg mL^−1^; tyrosol, 29.2 μg mL^−1^; rosarin, 68.8 μg mL^−1^; rosavin, 60.8 μg mL^−1^; and rosin, 104.8 μg mL^−1^. Injection volume: 10 μL; detection wavelength: 205 nm.

## Abbreviations

The following abbreviations are used in this manuscript:

HPLC	High-performance liquid chromatography
LOD	Limit of detection
LOQ	Limit of quantification
UV	Ultraviolet spectrum
Vis	Visible spectrum
ACN	Acetonitrile
H_2_O	Water
RSD	Relative standard deviation
R^2^	Coefficient of determination
*R*_s_	Chromatographic resolution
