# Supplementary material for: A Simplified and Solvent-Reduced HPLC-UV/Vis Method for the Simultaneous Determination of Major Marker Compounds in Rhodiola rosea L
Source: Molecules. 2026 Oct 18;31(20):3734. doi: 10.3390/molecules31203734 (PMC13707433; doi:10.3390/molecules31203734)
Supplement: Supplementary file 1 [file molecules-31-03734-s001.zip › molecules-4613884-supplementary.pdf]

Supplementary Material

A Simplified and Solvent-Reduced HPLC-UV/Vis Method for the Simultaneous Determination of Major Marker Compounds in *Rhodiola rosea* L.

Alexandra Stabl [1,*], Katalin Reich [1], Ivonne Wittmann [2], Sabine Wittmann [3], Özlem Özmutlu Karslioglu [4] and Sabine Grüner [1,*]

[1] Department of Bioengineering, University of Applied Sciences Weihenstephan-Triesdorf, Am Hofgarten 10, 85354 Freising, Germany
[2] Institute of Horticulture, University of Applied Sciences Weihenstephan-Triesdorf, Am Staudengarten 8, 85354 Freising, Germany; ivonne.wittmann@hswt.de
[3] Digital Agriculture, HEF World Agricultural Systems Center, Department of Life Science Engineering, TUM School of Life Sciences, Technical University of Munich, 85354 Freising, Germany; sabine.wittmann@tum.de
[4] Institute of Food Technology, University of Applied Sciences Weihenstephan-Triesdorf, Am Staudengarten 11, 85354 Freising, Germany; oezlem.oezmutlu@hswt.de
* Correspondence: alexandra.stabl@hswt.de (A.S.); sabine.gruener@hswt.de (S.G.)

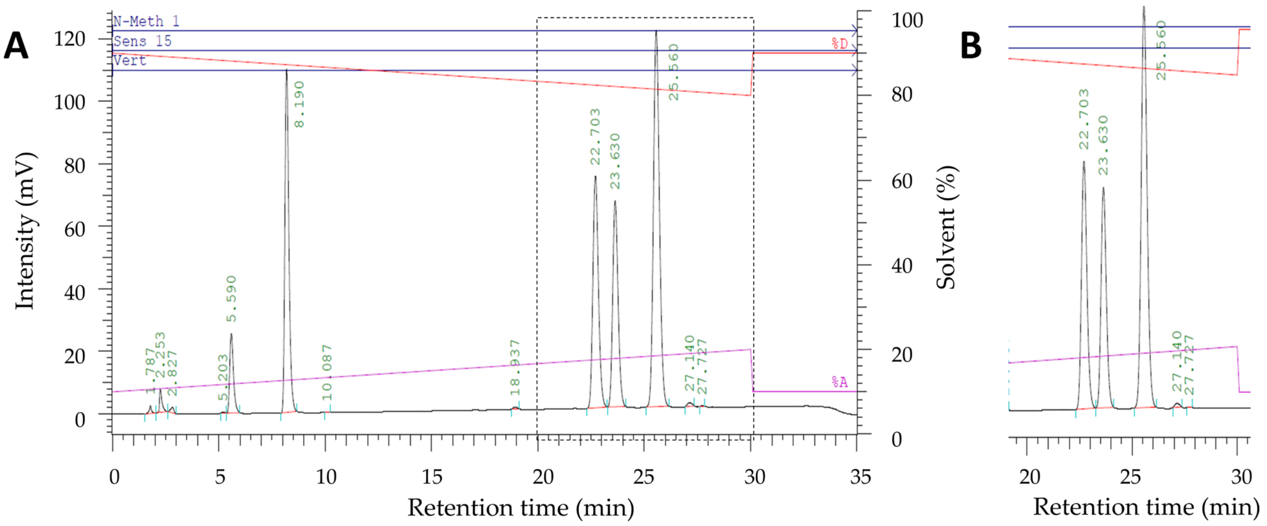

Figure S1. Chromatogram of a higher-concentration mixed reference standard: (A) complete chromatogram of the five target analytes and (B) enlarged view of the rosavin-containing region, highlighting the critical rosarin–rosavin peak pair. Analyte concentrations: salidroside, 84.0 μg mL^{-1}; tyrosol, 29.2 μg mL^{-1}; rosarin, 68.8 μg mL^{-1}; rosavin, 60.8 μg mL^{-1}; and rosin, 104.8 μg mL^{-1}. Injection volume: 10 μL; detection wavelength: 205 nm
